# Epidemiology of Frequent/Urgent Urination in Older Adults in China: A Multicenter, Cross-Sectional Study

**DOI:** 10.3389/fpubh.2021.669070

**Published:** 2021-09-07

**Authors:** Yiwen Zhang, Xiao-Dan Wang, Yehua Song, Ruiqiang Peng, Ting Tang, Miaoduan Li, Zhenzhen Yu, Yong Ji, Jianping Niu

**Affiliations:** ^1^Department of Neurology, The Second Affiliated Hospital of Xiamen Medical College, Xiamen, China; ^2^Tianjin Key Laboratory of Cerebrovascular and of Neurodegenerative Diseases, Department of Neurology, Tianjin Dementia Institute, Tianjin Huanhu Hospital, Tianjin, China; ^3^China National Clinical Research Center for Neurological Diseases, Department of Neurology, Beijing Tiantan Hospital, Capital Medical University, Beijing, China

**Keywords:** frequent urination, urgent urination, prevalence, risk factors, epidemiological survey

## Abstract

**Background:** Frequent/urgent urination is an event of multifactorial origin where involuntary leakage of urine occurs. Epidemiological study of this condition is of high importance due to its negative impact on the psychological, physical, and social well-being of the victims.

**Objective:** This cross-sectional study aimed to investigate the prevalence of frequent/urgent urination in older adults in China.

**Method:** In this study, a face-to-face questionnaire survey was conducted between April 2019 and August 2019 among 4,796 older adult populations in the communities of Tianjin jizhou and Xiamen jimei of China. Descriptive analysis, univariate regression, and all statistics were conducted in IBM SPSS v22. The count data were analyzed by chi-square test. *P* < 0.05 was considered statistically significant.

**Results:** In the total investigated population, the prevalence of frequent or urgent urination was found in 1,164 patients (24.3%) where 31.7% (664/2,097) were male patients and 18.7% (500/2,699) were female patients, having a male-to-female ratio of 1.7:1. The prevalence was higher in the 70- to 84-year-old group (men: 33.3–34.8%, women 19.5–20.8%), whereas it was relatively low in the 65- to 69-year-old group and in older adults over 85 years of age (men 28.8, 30.3%, women 16.7, 18.5%, respectively). In terms of the course of the disease, among the population aged 65 years and above, 17.3% men and 9.9% women had frequent urination/urgency lasting for 1–4 years; 5–9 years in about 4.5% population (7.4% men and 4.2% women); 10–19 years in 4.9% men and 2.3% women; and more than 20 years duration in 1.6% men and 1.9% women. On the severity scale, mild frequent/urgent urination was observed in 24.6% of men and 15.4% women of Chinese older adults. Moderate cases were observed in 6.3% of men and 2.9% of women, whereas severe cases were found in 0.8% men and 0.2% women. Benign prostatic hyperplasia (BPH)/hypertrophy was the main risk factor for frequent/urgent urination in Chinese older adult men (*P* < 0.001). Obesity, hypertension, diabetes, heart disease, anxiety, depression, constipation, and brain injury were the other risk factors for frequent/urgent urination in Chinese older adult men and women. The results of this survey showed that smoking or drinking habits did not increase the prevalence of frequent/urgent urination in Chinese older adults.

**Conclusions:** According to the results of this survey, the prevalence rate of frequent/urgent urination is high, and the course of the disease is long in Chinese older adults. BPH and depression, anxiety, and age-related chronic diseases increase the risk of frequent/urgent urination in Chinese older adults.

## Introduction

Frequent/urgent urination has been defined by the International Continence Society (ICS) as “any involuntary leakage of urine” and describes this as an event of multifactorial origin (1). Though this physiological disorder is not essential to the process of aging, it occurs more with advanced age and is, thus, believed to be one of the key syndromes in geriatrics (2–4). As the number of older adults increases, an increase in the occurrence of frequent/urgent urination has been documented (3, 5). Literature study describes various factors associated with the occurrence of this condition, including advanced age and female gender (6–8), ethnic groups, i.e., non-Caucasian, and low levels or lack of schooling (7, 9, 10). Different diseases have also been the key predictors for this phenomenon like prostatic hyperplasia (11), urinary tract infection (12), diabetes mellitus (13–15), arterial hypertension (14), cardiac diseases (5), stroke (6, 7, 13, 16), obesity (5), respiratory problems (16), cognitive deficit (16), depression (6), arthritis (12), rheumatism/arthrosis (17), history of falls (5, 17), functional limitations, dependence or limited mobility (5, 11, 16), comorbidities (18), and frailty (17). Moreover, lifestyle habits, like sedentarism, can also contribute to this disease (19). Association with other factors like polypharmacy, gynecological surgery, hysterectomies and menopause in women (19), a negative self-assessment of health status, and poor quality of life has also been documented as risk factors for frequent/urgent urination (18).

Epidemiological study of this disease is of high importance not only due to the high prevalence in older adults but also due to its negative impact on psychological, physical, and social aspects of the affectees (20). Frequent/urgent urination tends to induce changes in the routine life of the older adults, leading to social isolation due to loss of self-esteem and embarrassment, thus impairing their quality of life (21). Moreover, such humiliation prevents affected individuals from gaining diagnosis and professional help, making the disease more permanent (3). Though the symptoms of frequent urination are still omitted or underestimated due to the negative emotions and since the disease is considered as a part of the natural process of aging (3, 7, 22, 23), it is still reflected as the major healthcare complaint.

There are limited population-based epidemiological surveys for frequent/urgent urination in Chinese older adult population, especially in the Tianjin jizhou and Xiamen jimei regions of China. Lu et al. have conducted a study in premenopausal women in Wuhan to assess the prevalence and risk factors of urinary incontinence (UI) (24). Liu et al. have conducted a study in Shanghai to assess the prevalence and risk factors of frequent urination among Chinese women (25). In another population-based study, Ge et al. have attempted to assess the prevalence and risk factors of frequent urination in women in Beijing, China (26). This study aimed to investigate the prevalence, course, severity, and related risk factors of frequent/ urgent urination in Chinese older adult population in the Tianjin jizhou and Xiamen jimei regions of China. The report is as follows.

## Data and Methods

### Subjects and Survey Period

A face-to-face questionnaire survey was conducted between April 2019 and August 2019 among 4,796 Chinese older adults in the communities of Tianjin jizhou and Xiamen jimei of China. Inclusion of the subjects was based on the following: (1) Subject should have complete information on the questionnaire; (2) be aged ≥65 years and older; (3) agree to participate in the survey voluntarily and sign the informed consent. Exclusion criteria were as follows: (1) subjects who refuse to sign the informed consent and (2) respondents with incomplete questionnaire data.

There were 2,097 men and 2,699 women, aged 65–100 years, with an average age of 72.11 ± 5.92 years.

### Methods

Research methods: This study adopted the method of a cross-sectional survey, designed the questionnaire, trained the members and assistants of the research group, unified the survey methods and standards, and conducted a face-to-face questionnaire survey on the respondents with the assistance and coordination of community workers. The survey form was filled manually by the volunteers who agreed to take part in this survey, and in the case where volunteers could not write, they were assisted by the research team and assistants.

Survey content: The survey content included the age of the patient, gender, marital status, smoking history, drinking history, obesity, hypertension, heart disease, anxiety and depression, constipation, and brain injury (included craniocerebral trauma, hemorrhage, and infarction). For men, it also included the presence or absence of a history of benign prostatic hypertrophy (BPH).

Estimation of sample size: The epidemiological formula *n* = 400(1 – *P*)/*P* was used for the estimation of sample size, where *P* is the estimated prevalence. It has been reported that the average prevalence of frequent/urgent urination is 32.5 and 27.5% in rural and urban areas, respectively, in China (27). Based on this calculation, it was estimated that the sample size should be >1,055 in urban areas and >831 in rural areas.

Determination of frequent/urgent urination and assessment of morbidity: According to the definition of ICS, the criteria for determining frequent/urgent urination are as follows: (i) frequency of urination ≥ 8 times every 24 h and/or (ii) chief complaint of sudden occurrence of a strong urge to urinate by the patients (28). The assessment contents included the prevalence rate, duration of the disease, severity, and risk factors of urinary frequency/urgency in different gender and age groups. Among them, the criteria for determining the severity of frequency/urgency were as follows: mild, occasional frequency/urgency without medication; moderate, frequency/urgency with medication; severe, frequency/urgency with incontinence and/or incomplete bladder emptying, requiring intermittent catheterization; and extremely severe, frequency/urgency with incontinence that requires indwelling catheters.

### Statistical Treatment

Descriptive analysis, univariate regression, and all statistics were conducted in IBM SPSS v22. The count data were analyzed by chi-square test. *P* < 0.05 was considered to be statistically significant.

## Results

### Prevalence of Frequent/Urgent Urination

In this study, a total of 4,796 Chinese older adults were investigated in China. Among them, 1,164 people had frequent or urgent urination, with a prevalence rate of 24.3% (1,164/4,796). Among 1,164 patients, the prevalence rate of men was 31.7% (664/2,097) and that of women was 18.5% (500/2,699), with the 1.7:1 ratio of men to women, as shown in Table 1.

**Table 1:** Prevalence of frequent/urgent urination in Chinese older adults.

**Gender**	**Number of respondents (cases)**	**Number of patients (cases)**	**Prevalence (%)**
Male	2,097	664	31.7
Female	2,699	500	18.5
Total	4,796	1,164	24.3

*The chi-square statistic is 66.8427. The P-value is <0.00001*.

### Prevalence of Frequent/Urgent Urination at Different Age Levels

The prevalence of frequent/urgent urination was relatively low in the 65- to 69-year-old level and ≥ 85-year-old level, and higher in the 70- to 84-year-old level, regardless of men and women, as shown in Table 2.

**Table 2:** Prevalence of frequent/urgent urination in Chinese older adults.

**Age (years)**	**Gender**	**Number of respondents (cases)**	**Number of patients (cases)**	**Prevalence (%)**
65–69	Male	838	241	28.8
	Female	1,144	191	16.7
70–74	Male	578	195	33.7
	Female	792	159	20.1
75–79	Male	348	121	34.8
	Female	442	86	19.5
80–84	Male	213	71	33.3
	Female	236	49	20.8
≥85	Male	119	36	30.3
	Female	81	15	18.5

*The chi-square statistic for the above data is 7.1458. The P-value is 0.128379. The results are not significant at P < 0.05*.

### The Duration of Frequent/Urgent Urination

Among Chinese older adults, 0.47% men (10/2,097) and 0.18% of women (5/2,699) had frequent/urgent urination lasting <1 year; 17.3% of men (362/2,097) and 9.9% women (268/2,699) had 1–4 years duration of frequent urination; 7.4% of men (155/2,097) and 4.2% women (113/2,699) had 5–9 years duration; 4.9% men (103/2,097) and 2.3% women (63/2,699) had 10–19 years duration; and 1.6% men (34/2,097) and 1.9% of women (51/2,699) had more than 20 years duration of frequent/ urgent urination, as shown in Table 3.

**Table 3:** The duration of frequent/urgent urination in Chinese older adults.

	**Male (cases)**	**Prevalence (%)**	**Female (cases)**	**Prevalence (%)**
<1 year	10	0.47	5	0.18
1–4 years	362	17.3	268	9.9
5–9 years	155	7.4	113	4.2
10–19 years	103	4.9	63	2.3
≥20 years	34	1.6	51	1.9

*The chi-square statistic is 18.9943. The P-value is 0.000788. The results are significant statistically at P < 0.05*.

### The Severity of Frequent/Urgent Urination

According to the survey, 24.6% of the men (515/2,097) and 15.4% of the women (415/2,699) had mild frequent/urgent urination; 6.3% of the men (132/2,097) and 2.9% of the women (79/2,699) had moderate frequent/urgent urination; and 0.8% of the men (16/2,097) and 0.2% of the women (6/2,699) had severe frequent/urgent urination. There was one very severe frequent/urgent urination patient among the 2,097 male older adults interviewed, with a prevalence rate of 0.05% (1/2,097), and there was no very severe frequent/urgent urine patient among the 2,699 female older adults interviewed, with a prevalence rate of 0 (0/2,699), as Table 4 shows.

**Table 4:** The severity of frequent/urgent urination in Chinese older adults.

	**Male**	**Female**
Number of mild patients (cases)	515	415
Prevalence (%)	24.6	15.4
Moderate patients (cases)	132	79
Prevalence (%)	6.3	2.9
Severe patients (cases)	16	6
Prevalence (%)	0.8	0.2
Very severe patients (cases)	1	0
Prevalence (%)	0.05	0

*The chi-square statistic is 74.5. The P-value is < 0.00001. The results are significant at P < 0.05*.

### Relationship Between Frequency/Urgency and Risk Factors

The survey shows that the prevalence of frequent/urgent urination in patients with BPH/hypertrophy is significantly higher (*P* < 0.001) in Chinese older adult men. For Chinese older adults, regardless of gender, the number of patients with risk factors of obesity, hypertension, diabetes, heart disease, anxiety and depression, constipation, and brain injury increased. The prevalence difference between those with and without the above risk factors was statistically significant. The results of this survey showed that smoking or drinking habits did not increase the prevalence of frequent/urgent urination in Chinese older adults, as shown in Tables 5, 6. Logistic multivariate regression analysis of risk factors for frequent/urgent urination in both men and women is given in Table 7.

**Table 5:** Risk factors of frequent/urgent urination in Chinese older adult men.

**Risk factor**	**Patients with frequent/urgent urination**	**Patients without frequent/urgent urination**	***X*^2^**	***P***
**BPH**				
Yes	273	72	429.966	<0.001
No	391	1,361		
**Obesity**				
Yes	22	20	8.482	0.005
No	642	1,413		
**Smoking**				
Yes	388	789	2.098	>0.10
No	276	644		
**Drinking**				
Yes	340	705	0.731	>0.25
No	324	728		
**Hypertension**				
Yes	374	687	12.562	<0.001
No	290	746		
**Diabetes**				
Yes	109	149	15.230	0.000
No	555	1,284		
**Heart disease**				
Yes	110	165	10.164	0.000
No	554	1,268		
**Anxiety and depression**				
Yes	14	12	5.986	<0.05
No	650	1,421		
**Constipation**				
Yes	159	155	61.434	0.000
No	505	1,278		
**Brain injury**				
Yes	148	252	6.503	<0.025
No	516	1,181		

**Table 6:** Risk factors of frequent/urgent urination in Chinese older adult women.

**Risk factor**	**Patients with frequent/urgent urination**	**Patients without frequent/urgent urination**	***X*^2^**	***P***
**Obesity**				
Yes	45	105	13.855	0.000
No	455	2,094		
**Smoking**				
Yes	30	111	0.746	>0.25
No	470	2,088		
**Drinking**				
Yes	26	90	0.865	>0.25
No	474	2,109		
**Hypertension**				
Yes	333	1,239	17.620	0.000
No	167	960		
**Diabetes**				
Yes	95	307	8.161	<0.005
No	405	1,892		
**Heart disease**				
Yes	146	367	41.418	0.000
No	354	1,832		
**Anxiety and depression**				
Yes	27	49	14.975	0.000
No	473	2,150		
**Constipation**				
Yes	168	338	88.869	0.000
No	332	1,861		
**Brain injury**				
Yes	97	278	15.551	0.000
No	403	1,921		

**Table 7:** Logistic multivariate regression analysis of risk factors for frequent/urgent urination in both men and women.

**Risk factor**	***B***	**SE**	**Wald**	***P***	**OR**
Obesity	0.558	0.155	12.99	0.000	1.7
Hypertension	−1.134	0.034	1,135.3	0.000	0.322
Diabetes	−1.134	0.034	1,135.3	0.000	0.322
Heart disease	−1.134	0.034	1,135.3	0.000	0.322
Anxiety/Depression	−1.134	0.034	1,135.3	0.000	0.322
Constipation	−1.134	0.034	1,135.3	0.000	0.322
Brain injury	−1.134	0.034	1,135.3	0.000	0.322

*Since smoking and drinking were non-significant in univariate analysis, these factors were eliminated*.

## Discussion

According to the epidemiological investigation, apart from urinary tract infection (UTI) and other obvious pathological causes, the incidence of overactive bladder (OAB) is 7–27% in men and 9–43% in women and this incidence tends to increase with age (29, 30). Studies have shown that the prevalence of OAB without urge urinary incontinence (UUI) in men under 45 years of age increased from 8.5 to 21.8% after the age of 55 years, while the prevalence of OAB without UUI in women increased gradually with age and reached the highest level after the age of 44 years (30). The epidemiological analysis from China showed that the overall incidence of OAB excluding urinary tract infection and other obvious pathological changes was 6.0%, and the incidence gradually increased with the aging of the population (31). The epidemiological study of frequent/ urgent urination is of paramount importance because it seriously interferes with the social functions of the patients, affects their quality of life, and brings a heavy economic burden to their family and society. According to the statistics, the medical expenses caused by OAB in the United States are as high as several billion dollars (32).

In this study, the overall prevalence rate of the disease among Chinese older adults was 24.3%, among which the prevalence was 31.7% in men and 18.7% in women and the ratio of men to women was 1.7:1. Previous studies in regions of Europe and North America found high prevalence rates of frequent urination ranging from 10.8 to 17.4% for both men and women (33–35). The prevalence of frequent/urgent urination in older adult men was higher than that in women, which was considered to be related to the high prevalence of BPH /hypertrophy in older adult men. This study shows that the prevalence of frequent/urgent urination in Chinese older adults was higher at 70–84 years old level, while it was relatively low at 65–69 and over 85 years old levels, regardless of gender. The prevalence of frequent/urgent urination was higher in the 70–84 years age level than in the 65–69 years age level, which was in line with the rule that the prevalence of this condition increases with age. The overall decreased prevalence of frequent and urgent urination in the age level of 85 years and above may be related to the relatively good healthcare services provided to this age group. Moreover, the duration of disease in the Chinese senior population was varying ranging from 1–4, 10–19, to more than 20 years. Regarding the severity of the disease, 24.6% of men and 15.4% of women had mild frequent/urgent urination, 6.3% of men and 2.9% of women had moderate frequent/urgent urination, and 0.8% of men and 0.2% of women had severe frequent/urgent urination. The above data show that frequent/urgent urination not only has a high prevalence in the older adult population in China but also has a long course of the disease. Some patients were more seriously ill, suggesting that frequent/urgent urination is seriously affecting the quality of life of the older adults in China, which should be paid attention to by relevant decision-making departments and researchers.

According to the results of this study, for Chinese older adult men, the prevalence of frequent urination in patients with BPH/hypertrophy is significantly increased, which is consistent with the results of the existing research studies (30, 36, 37). BPH is the most common cause of lower urinary tract symptoms (LUTS) in older adult men (30, 36, 37). Therefore, reasonable and effective treatment of BPH/hypertrophy is very important to reduce the prevalence of the disease in older adult men. This study shows that obesity, hypertension, diabetes, heart disease, and brain injury (including brain trauma, cerebral hemorrhage, and cerebral infarction) are common risk factors for frequent/urgent urination of older adults in China. As age increases, the incidence of metabolic syndrome-related diseases such as obesity, diabetes, and hypertension, and heart disease and cerebrovascular disease increases (38, 39). These chronic diseases may lead to an obstruction in the reflex pathway of urination through atherosclerosis; chronic pelvic ischemia; central or peripheral nerve ischemia; metabolic lesions; and local muscle, nerve, and epithelial mucous membrane lesions of the bladder, resulting in frequent/urgent urination and other clinical symptoms. Therefore, targeted prevention and control of common chronic diseases such as obesity, hypertension, diabetes, heart disease, and cerebrovascular disease will help to reduce the prevalence of frequent/urgent urination in older adults. Anxiety and depression were also observed as risk factor for frequent and urgent urination in older adults in China, and the results of this study are similar to those of other age groups (30, 40). The possible mechanisms underlining the association of anxiety with this disease may be the decreased expression of 5-hydroxytryptamine in patients with anxiety and depression, and the weakened inhibition of the urinary reflex pathway. Meanwhile, the sympathetic system is activated and norepinephrine expression is increased, which leads to the change of bladder physiological activity and frequent urination. Constipation was also a risk factor associated with frequent/urgent urination in the Chinese older adult population. Previous studies conducted in children and middle-aged women have also reported a positive correlation between constipation and frequent urination (41, 42). The possible mechanisms involved in the association of constipation with frequent urination are that the rectum and bladder are anatomically close, the motor nerves that control the functions of the two originate from the same sacral nerve (S2–S4), and they may have the same pathogenic factors and lesion process. In addition, the pelvic floor muscle damage caused by long-term overexertion in defecation and the resulting dysuria may be one of the reasons for the increased prevalence of frequent/urgent urination in patients with constipation (43). It is observed in this study that smoking or drinking habits did not increase the prevalence of frequent/urgent urination in Chinese older adults. This is contrary to the theory that smoking induces atherosclerosis and ischemic injury of micturition reflex pathway, and alcohol damages the micturition center or peripheral nerve, both of which could lead to dysuria and increase the prevalence of frequent and urgent urination (44, 45). It is speculated that the reason is that the sample size included in this study is not large enough, and more widespread studies are needed for a clear conclusion about the association of smoking or drinking habits with frequent urination.

In conclusion, the prevalence of frequent/ urgent urination was found to be higher and the course of the disease was longer in the older adults, and some patients were seriously ill, which is seriously affecting the social life of the older adults in China. In terms of risk factors, anxiety, depression, constipation, age-related chronic diseases such as BPH/hypertrophy, hypertension, diabetes mellitus, cardiovascular, and cerebrovascular diseases increase the risk of frequent/ urgent urination in older adults. Of course, the conclusions of this study are drawn from multicenters in Mainland China, which may not reflect the situation of all regions in China. Future studies will further expand the geographical coverage of the study and increase the sample size of the older adult population to more accurately reflect the prevalence of frequent/urgent urination of the older adults in China and to promote the health of the older adults. Second, our study is more representative of the Tianjin jizhou and Xiamen jimei region, which may differ from the findings in other regions of China, with some regional limitations. Moreover, this study was cross-sectional and because the exposure and outcome were simultaneously assessed, there was generally no evidence of a temporal relationship between exposure and outcome; more samples and multilevel clinical epidemiological surveys are needed.

Considering the continued aging of Chinese population, frequent/urgent urination is bound to become an important health problem affecting the health of older adults in China. It is necessary to develop a comprehensive intervention plan for reproductive health services covering women and men of all ages. Anxiety in the older adult population has to be reduced *via* implementing policies that contribute to the reduction of anxiety in the older adult population. The findings suggested that for older people in the studied communities and in overall China, educational and awareness programs should be conducted to spread awareness and understanding about the early symptoms of the disease. Ways to enhance support and acceptable forms of specific content knowledge, process measures, and regulatory enforcement require additional exploration.

## Data Availability Statement

The generated datasets will be available only by request to the corresponding author. Requests to access the datasets should be directed to Corresponding authors: Yong Ji, Email: jiyongusa@126.com; Jianping Niu, Email: 549872685@qq.com.

## Ethics Statement

The studies involving human participants were reviewed and approved by the Tiantan Research Ethics Committee (2019-40). The patients/participants provided their written informed consent to participate in this study.

## Author Contributions

YZ and X-DW carried out all the research. YS prepared the initial manuscript. RP analyzed the data. TT and ML finalized the manuscript. ZY completed the final formatting and review of the manuscript. YJ and JN supervised the whole study. All authors contributed to the article and approved the submitted version.

## Funding

This work was supported by the National Key Research and Development Program of China (grant number 2016YFC1306305) and the Scientific Research Project of Tianjin Nursing Association (grant number tjhlky2020YB05).

## Conflict of Interest

The authors declare that the research was conducted in the absence of any commercial or financial relationships that could be construed as a potential conflict of interest.

## Publisher's Note

All claims expressed in this article are solely those of the authors and do not necessarily represent those of their affiliated organizations, or those of the publisher, the editors and the reviewers. Any product that may be evaluated in this article, or claim that may be made by its manufacturer, is not guaranteed or endorsed by the publisher.
